# Diamond Blackfan Anemia: a Tertiary Care Center Experience

**DOI:** 10.4084/MJHID.2013.039

**Published:** 2013-06-03

**Authors:** Avinash Kumar Singh, Nita Radhakrishnan, Tulika Seth, Pravas Mishra, Manoranjan Mahapatra, Haraprasad Pati

**Affiliations:** Department of Hematology, All India Institute of Medical Science, New Delhi, India

## Abstract

Diamond Blackfan Anemia (DBA) is a rare hypoplastic anemia that presents in infancy with macrocytic anemia and reticulocytopenia. It is a ribosomopathy with autosomal dominant inheritance.

In our series of 10 patients with DBA, congenital malformations were observed in 50% of the cases. Age at symptom onset ranged from 0–12 months. Age at diagnosis ranged from 4 months to 96 months. Male: female ratio was 9:1. Response to prednisolone was observed in 4 out of the 10 patients (either during initial treatment or during re-challenge). Response to cyclosporine was found to be poor. Bone marrow transplantation was successful in attaining remission in one patient. Malignancies were not reported in any patient possibly due to a short follow up period.

## Introduction

Diamond Blackfan anemia (DBA) is a rare congenital hypoplastic anemia that usually presents with progressive pallor in infancy. It is characterized by normocytic to macrocytic anemia, reticulocytopenia and a normocellular marrow with markedly decreased or absent erythroid precursors. White cell count is normal or slightly decreased where as platelet count is normal to slightly increased. Congenital malformations are seen in up to 50% of cases and there is an increased risk of malignancy.^1^ It is a ribosomopathy; caused by genetic mutations that affect ribosome synthesis. This results in a cellular defect of erythroid precursors, causing apoptosis and erythropoietic failure. About 45% of cases are familial; usually displaying autosomal dominant inheritance with variable penetrance.^2^ There are limited publications regarding presentation and response to treatment in DBA patients from India.^3^–^7^ We report 10 such patients diagnosed and followed up at our center.

## Methods

All patients were evaluated with a detailed history, complete blood count and bone marrow examination. Vitamin B12 and folate deficiencies were excluded prior to performing bone marrow study. Diagnostic criteria for DBA discussed by Vlachos et al were applied in our analysis.^8^ Age less than 1 year at onset of disease, macrocytic anemia without any other cytopenias, reticulocytopenia and normal marrow cellularity with a paucity of erythroid precursors were considered as diagnostic of DBA. Parvovirus study were performed only in few cases due to monetary constraints.

All patients were started on prednisolone at a dose of 2mg/kg/day as a single dose in the morning. Prednisolone was tapered very slowly once response to therapy was noted in the form of rise in hemoglobin to normal range. Age appropriate levels of hemoglobin were considered while assessing response to therapy. If no response was observed within 6–8 weeks, steroids were tapered and stopped. In patients who failed prednisolone therapy, cyclosporine was given at 5 mg/kg/day in two divided doses. Kidney and liver functions were monitored during cyclosporine therapy. Bone marrow transplant was done in one patient who had a matched sibling donor and who failed initial therapy. Treatment response was assessed as per criteria used by the DBA registry.^9^ Remission or complete response (CR) was defined as hemoglobin at a physiologically acceptable level (>11.5 gm/dl in our series) that was maintained for more than 6 months independent of any therapy or transfusions. Patients who were transfusion independent with less than acceptable level of hemoglobin was defined as partial response (PR) and those who did not respond were labeled non-responders (NR).

## Case Series

A total of 10 children (9 male and 1 female) were diagnosed at our center with DBA. The age of symptom onset was less than 12 months in all cases. The age of presentation to our center ranged from 4 months to 96 months. All patients presented with history of pallor since infancy requiring 2 to 6 packed RBC transfusions prior to presentation. 2 patients who were siblings were born after consanguineous marriage. None of the patients were small for gestational age at birth. All our patients fulfilled the diagnostic criteria suggested by DBA consensus statement.^8^

On examination all had pallor and mild hepatomegaly. Abnormal phenotypic features seen were present in 6 patients (60%). The congenital abnormalities seen included abnormal thumbs (hypoplastic thumbs and clinodactyly) in 6 patients (60%), triangular facies in 5 (50%)and microcephaly, low set ears, low posterior hairline, wide spaced teeth, flat nasal bridge, wide spaced toes in 1 patient each (10%). Growth failure was noted in 3 patients (30%), delayed bone age in 2(20%) and wide spaced nipples in 1 patient (10%). Other systems were normal on examination. Patient characteristics have been detailed in Table 1.

Investigations revealed hemoglobin ranging from 2.1 to 11 g/dL with a corrected reticulocyte count of <1%. The peripheral smear showed normocytic to macrocytic and normochromic anemia with mild anisocytosis. Leucocyte counts were normal in all patients; however, platelet counts were high in 3 patients. All patients had elevated serum iron levels with transferrin saturation ranging between 20 to 40%, except one who had co-existing iron deficiency anemia at presentation. In this patient, anemia persisted after correction of iron deficiency. Skeletal surveys and ultrasonography of the abdomen ruled out other congenital anomalies. Stress cytogenetics was done for 1 patient, which was normal. The diagnosis was confirmed on bone marrow aspiration and trephine biopsy, which showed marked erythroid hypoplasia with elevated myeloid: erythroid ratio ranging from 15:1 to 20:1. Megakaryopoiesis and myelopoiesis were normal in all.

## Treatment and follow up

### Response to treatment

As described earlier, the criteria for remission were adopted from the DBA registry data. Out of our series of 10 patients in whom prednisolone was started as initial therapy at a dose of 2mg/kg/day, one patient attained complete response within 6 weeks. Prednisolone was tapered and stopped in this patient and he remained transfusion free for the last 13 months. 3 patients attained partial response within 6–8 weeks of prednisolone. On tapering prednisolone, one patient relapsed and in him it was restarted at 2mg/kg/day. He attained partial response within next 8 weeks and is transfusion independent on a maintenance dose of prednisolone (<0.5mg/kg/day) for the last 7 years. In the rest 2, CSA has been started and both remain prednisolone dependent with no change in their response status.

The remaining 6 patients did not show any response to prednisolone during the initial 8 weeks. 3 out of 6 patients were started on CSA, but failed to respond. One of these patients underwent matched sibling bone marrow transplant at another center and has remained symptom free for the last 8 years. The other 2 patients remain transfusion dependent. In the remaining 3 patients, one was lost to follow up, and the rest 2 have been on steroids and are still transfusion dependent.

### Follow up

To conclude, at a median follow up of 24.5 months (4 months–11 years), out of 10 patients, response to steroids (either complete or partial, either during initial therapy or during re-challenge) has been shown by 4 patients (40%). Our experience with CSA has been poor as none of the patients showed any benefit with it. The patient who underwent BMT is undergoing regular phlebotomy for iron overload and is on treatment for chronic hepatitis C and hypothyroidism. Out of the 4 patients who are transfusion dependent, 2 are on chelation therapy (Deferasirox) for iron overload.

## Discussion

**Diagram 1:**
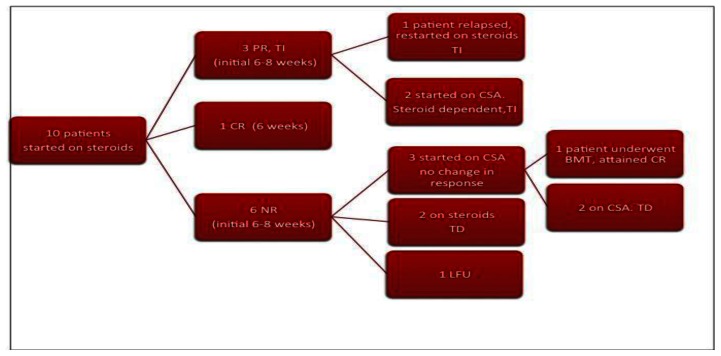


In 1938, Diamond and Blackfan described a congenital red cell aplasia characterized by decreased or absent bone marrow erythroid precursor cells, severe anemia and reticulocytopenia.^10^,^11^ Diamond-Blackfan anemia (DBA) is a heterogeneous disorder in which several pathophysiologic mechanisms result in disturbed erythropoiesis at various stages along the erythroid differentiation pathway.^12^ DBA is frequently associated with congenital abnormalities. The prevalence of genitourinary and cardiac anomalies may be underestimated when abdominal, pelvic and cardiac sonography is not routinely performed. The prevalence of craniofacial and limb anomalies in our series is comparable to those in published literature. None of our patients had renal or cardiac anomalies. Low birth weight although reported in 25% cases in other series, was seen in none of our patients. Up to 6% frequency of malignancy has been reported in other cohorts of patients with DBA.^8^ In our series, none has been observed so far probably because of short follow up. (Diagram 1)

Steroids remain the mainstay of therapy in DBA. Up to 80% patients are found to respond to an initial course of corticosteroids. Steroids are generally started once the hemoglobin level post transfusion is between 8–10gm/dl. This level is high enough to give adequate time to assess steroid response before next transfusion and low enough to prevent erythroid suppression.^8^ Once a patient responds, then very slow tapering has been advised to avoid the risk of overshooting the minimal effective dose in the patient. Inter current infections may cause fall in hemoglobin and require transfusion support.

Most registries report that 40% of patients continue being transfusion dependent after having failed steroid therapy. 20% of patients attain remission as defined as transfusion independent and on no medication.^8^,^13^ The rest (40%) continue as steroid dependent. Another series reports up to 80% initial response to steroids have been observed. 17% were nonresponsive and 4% of patients are never treated with steroids. Nearly half of the patients develop cushingoid features, 22% and 12% of patients respectively developed pathologic fractures and cataracts.^14^ In our series, remission has been observed in 2 patients (20%); one after steroid therapy and another after bone marrow transplantation. 3 patients are steroid dependent (30%) and 4 are transfusion dependent (40%). Response to initial steroids (complete and partial) was observed only in 4 cases (40%).

Several therapies like high dose methylprednisolone, interleukin-3, erythropoietin, androgens tried previously have not been found useful. Favorable response has been seen with leucine as well as valproic acid in some studies. However they are anecdotal and larger studies are required before considering them as standard therapy.^8^

## Figures and Tables

**Table 1:** Clinical Features and Lab Findings at presentation

	Case 1	Case 2	Case 3	Case 4	Case 5	Case 6	Case 7	Case 8	Case 9	Case 10
Age at presentation (months)	24	12	12	60	12	10	48	96	24	4
Age of 1st symptom (months)	12	10	10	12	8	Since birth	Since birth	Since birth	10	Since birth
Gender	Male	Female	Male	Male	Male	Male	Male	Male	Male	Male
Pallor	Present	Present	Present	Present	Present	Present	Present	Present	Present	Present
Congenital anomalies	Absent	Absent	Present	Present	Absent	Absent	Present	Present	Present	Present
Hepatomegaly	Yes	Yes	Yes	Yes	Yes	Yes	Yes	Yes	Yes	Yes
Splenomegaly	No	No	No	No	No	No	No	No	No	No
Hemoglobin (g/dL)	5.3	5.2	7.4	4.9	5.9	3	2.1	5.8	11	6
Platelet count (Lakhs/cmm)	3.36	6,0	3.0	2.6	6.0	6.6	4,4	2.5	2.1	3
MCV (fL)	91	98	92	90	84	92	99	100	98	100
Reticulocyte	0%	0.1%	0.5	0.5	0.8	0.8	0.7	0.1	0	0.9
HbF (%)	2.5	2.0	6	6	1	7	5	4	3	2
Transferrin saturation (%)	20	40	30	35	40	40	34	24	20	22
M:E ratio in bone marrow	16:1	16.:1	18:1	17:1	15:1	18:1	15:1	18:1	20:1	18:1
Family history	neg	neg	pos	pos	neg	neg	Neg	neg	neg	neg
